# Transformative Effect of Li Salt for Proactively Mitigating Interfacial Side Reactions in Sodium-Ion Batteries

**DOI:** 10.1007/s40820-025-01742-z

**Published:** 2025-04-21

**Authors:** Jooeun Byun, Joon Ha Chang, Chihyun Hwang, Chae Rim Lee, Miseung Kim, Jun Ho Song, Boseong Heo, Sunghun Choi, Jong Hyeok Han, Hee-Jae Jeon, Beom Tak Na, Youngjin Kim, Ji-Sang Yu, Hyun-seung Kim

**Affiliations:** 1 Advanced Batteries Research Center, Korea Electronics Technology Institute, 25, Saenari-Ro, Seongnam, 13509 Republic of Korea; 2 Analysis and Assessment Research Group, Research Institute of Industrial Science and Technology, Cheongam-Ro 67, Pohang, 37673 Republic of Korea; 3 Department of Battery Convergence Engineering, Kangwon National University, 1, Kangwon-Daehakro, Chuncheon-Si, Kangwon 24341 Republic of Korea; 4 Department of Mechanical and Biomedical Engineering, Kangwon National University, 1, Kangwon-Daehakro, Chuncheon-Si, Kangwon 24341 Republic of Korea; 5 Department of Smart Health Science and Technology, Kangwon National University, 1, Kangwon-Daehakro, Chuncheon-Si, Kangwon 24341 Republic of Korea; 6 Research Institute of Intelligent Manufacturing and Materials Technology, Korea Institute of Industrial Technology, Gaetbeol-Ro 156, Incheon, Republic of Korea

**Keywords:** Lithium salt, Electrolyte, Solid electrolyte interphase, Sodium-ion batteries, Additive

## Abstract

The robust respective formations of a solid electrolyte interphase (SEI) and pillar at the surfaces of hard carbon and O3-type positive electrodes are the consequences of integrating LiPF_6_ salt into a sodium-ion battery electrolyte that considerably strengthens both interfaces of positive and negative electrodes. The improvement of cycle performances due to the formation of highly passivating SEI on the hard carbon electrode is induced by the alternated solvation structure following the addition of Li salt, which inhibits sodium-ion and electron leakage from further electrolyte decomposition. The SEI with incorporated Li is less soluble than Na-based SEI, and the passivation ability of the initially formed SEI can thus be well preserved. Conversely, the gas evolution caused by oxygen release is reduced considerably by the marginal surface intercalation of Li ions at the surface of the O3-positive electrode. Additionally, the LiF layer that forms on the O3 surface diminishes additional deterioration of the electrolyte after formation. Compared with the fluoroethylene carbonate additive that is typically applied, a simultaneously strengthened interface yields major improvements in capacity retention.

**Supplementary Information:**

The online version contains supplementary material available at 10.1007/s40820-025-01742-z.

## Highlights

- LiPF_6_ integration into sodium-ion battery electrolytes strengthens solid electrolyte interphase (SEI) film and stabilizes O3 electrode surfaces, enhancing cycleability with 92.7% at 400 cycles.
- Li-based SEI exhibits reduced solubility, effectively suppressing sodium-ion and electron leakage, and mitigating electrolyte decomposition on hard carbon electrode.
- The formation of Li-ion pillars on O3-type electrode surfaces significantly reduces oxygen release and electrolyte degradation, resulting in improved capacity retention.

**Supplementary Information:**

The online version contains supplementary material available at 10.1007/s40820-025-01742-z.

## Graphical abstract

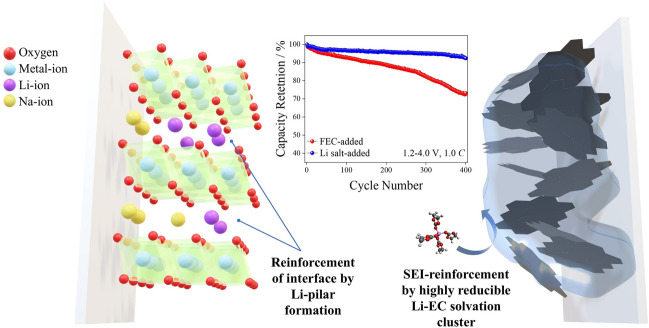

## Introduction

The substitution of charge carriers by sodium ions ensures the natural abundance of material resources. Hence, the supply of minerals can be stabilized, thereby delivering financially stable chemistry, resulting in large-scale energy storage and device-compatible, sodium-ion batteries (SIBs) [1–14]. The most widely used SIB chemistry is based on the hard carbon and layered transition metal oxide system [15–21]; therefore, the initially injected electrolyte is decomposed from the limited electrochemical stability window of the typical carbonate-based electrolytes [22–29]. From this decomposition, interphase formation occurs on the surface of the electrode in SIBs; however, the dominant decomposition characteristics of electrolytes in SIBs are considerably different than those of lithium-ion batteries (LIBs). The standard reduction potential of the Na/Na^+^ redox couple is 0.33 V higher than that of Li/Li^+^ redox couple; thus, the overpotential necessary to deliver the reduction current of the electrolyte, which expresses the kinetics of the formation of the solid electrolyte interphase (SEI), is not sufficient at identical voltage swing range [25, 30]. This results the thermally vulnerable performances of SIBs due to the insufficient deposition of SEI film on hard carbon electrode at SIB than that of LIBs. Furthermore, the number of solvated solvents is higher in SIBs compared with LIBs, and the reducibility of the solvated cation cluster is thus highly decreased [30, 31]. It is because the positive charge density of solvation cluster is decreased with the increment of solvation, which leads less reducible environment of electrolyte. Consequently, the formation of SEI in the SIBs is thermodynamically less favorable than in LIBs from thermodynamically and kinetically [30]. Moreover, formed sodium-ion-based SEI is more soluble in electrolytes compared with the SEI in the LIBs, implying that the chemical stability of SEI is insufficient for long-term operations [25, 32–34]. In layered structures, intercalation and de-intercalation of sodium ions severely degrade the interface because of the larger ionic radius of the sodium ion compared with that of the lithium ion [15–18]. The interfacial degradation and subsequent particle rupture of the O3-type positive electrode ensue gas evolution and rapid capacity degradation [1, 15–18], and thus, the positive electrode–electrolyte interface should be reinforced to improve the cycleability of SIBs. In brief, the simultaneous reinforcement of both interphases on the positive and negative electrodes should be conducted with distinct chemical species from the electrolyte additives. Typically, fluoroethylene carbonate (FEC), organic species, and sodium salts are added to the electrolyte to mitigate interfacial failure [35–39]; however, the present electrolyte composition is insufficient and unable to attain long-term cycle performances [40].

In this study, lithium hexafluorophosphate (LiPF_6_) was marginally added to modulate the interfacial side reactions in the most promising configuration, that is, hard carbon and O3 layered oxide-based SIBs. The addition of lithium salts in the SIB electrolyte alternates the local solvation structure with decreased lithium-ion solvation, indicating that a more reducible electrolyte is formed with lithium salts. SEI can be readily formed on the hard carbon electrode with the addition of LiPF_6_; additionally, the lithium-containing SEI is less soluble than Na-containing SEI components. Consequently, SEI can be fortified with the addition of LiPF_6_. In contrast, the slight surface doping of the O3-positive electrode from the addition of a lithium salt is performed during the initial formation; thus, the release of oxygen species and the deterioration of the surface can be mitigated; consequently, the gas evolution and further thick surface film deposition subsequently decreases. The alleviated failure from the addition of LiPF_6_ salt can suppress additional sodium/electron consumption after formation, implying that a substinatial cycle performance improvement is possible with hard carbon/O3-type layered oxide-based SIB.

## Experimental Section

### Electrolyte Formulation

0.5 wt% FEC (Soulbrain), 0.2 M LiPF_6_ (Chunbo), and 0.2 M lithium difluoro(oxalato)borate (LiODFB) were added to 1.0 M NaPF_6_ EC/DMC (1:1 = v/v, Dongwha Electrolyte), respectively. This mixture was prepared in an Ar-filled glove box and stirred overnight. The ionic conductivities of electrolytes were measured at 22 °C with a conductivity meter (Oakton CON 6 +).

### Electrode Preparation

A pouch cell was designed with a negative/positive electrode capacity ratio (N/P ratio) of 0.88. The pouch cell was assembled by stacking the positive electrode, a polyethylene separator, and the negative electrode. The negative electrode was prepared by using the slurry casting method, with 80:10:10 (wt%) of hard carbon (Kuraray):conductive carbon (TIMCAL, Super P):poly(vinylidene fluoride) (Kureha, KF-1100) in *N*-methyl-2-pyrrolidone (Sigma-Aldrich) on a 15 μm Al current collector. The electrode was pre-dried at 80 °C for 3 h and then dried under vacuum at 120 °C for 12 h. The hard carbon electrode had a mass loading of 8.0 mg cm^−2^ and an electrode density of 1.0 g cc^−1^. The positive electrode was 90:5:5 (wt%) O3-type NaNi_1/3_Fe_1/3_Mn_1/3_O_2_ (BTR):conductive carbon (TIMCAL, Super P):poly(vinylidene fluoride) (Kureha, KF-1100) in *N*-methyl-2-pyrrolidone (Sigma-Aldrich) on a 10 μm Cu current collector. The O3-type positive electrode had a mass loading of 15.01 mg cm^−2^ and an electrode density of 2.5 g cc^−1^. The drying process was performed was same as that used for the negative electrode.

### Coin and Pouch Cell Fabrication

The coin half-cell evaluation was performed with stacked electrode with sodium metal chip/glass filter/O3-type positive electrode and hard carbon electrode, respectively. After injecting the electrolyte and the additive-added electrolyte in 2032 coin cell, the sodium half-cell was aged for 24 h at room temperature to soak electrolyte. After aging, three cycles of the 0.1 *C* CC-CV (with 0.05 *C* cutoff) charge and followed 0.1 *C* CC discharge was performed for formation process with voltage range of 2.0–4.0 V (vs. Na/Na^+^) and 0.0–1.5 V (vs. Na/Na^+^) for O3-type positive electrode and hard carbon electrode, respectively, at room temperature. The 0.5 *C* CC-CV (with 0.05 *C* cutoff) charge and 0.5 *C* CC discharge was performed for room temperature cycling with voltage range of 2.0–4.0 V (vs. Na/Na^+^) and 0.0–1.5 V (vs. Na/Na^+^) for O3-type positive electrode and hard carbon electrode, respectively.

After injecting the electrolyte and the additive-added electrolyte, the cell was pressed to 1.0 MPa using a jig to evaluate its electrochemical cycling performance. Before electrochemical characterization, the pouch cell was rested for 24 h at room temperature.

The pouch cell formation was performed with a CC-CV charge of 0.1 *C* (with a 0.05 *C* cutoff) and with a CC discharge of 0.1 *C*, over a voltage range of 2.5–4.0 V. After formation, the cycleability was evaluated at room temperature with a CC-CV charge of 1.0 *C* (with a 0.05 *C* cutoff) and a CC discharge of 1.0 *C*, over a voltage range of 1.2–4.0 V.

Rate capability of pouch cell was evaluated with the range of 1.2–4.0 V. The *C*-rate for charging was constant with 0.5 *C*, and the discharging currents were varied with 0.1 *C*, 0.3 *C*, 0.5 *C*, 1.0 *C*, and 2.0 *C*, respectively.

### Spectroscopic and Morphological Analysis

X-ray photoelectrospectroscopy (XPS, Thermo Fisher) and scanning electron microscopy (SEM, JEOL) analysis were performed in a dry room. The cycled negative electrode was disassembled and dried under vacuum conditions for 12 h after rinsing with EMC. The positive and negative electrodes were dismantled at a state-of-charge of 0 after the 400 cycles. Time-of-flight secondary ion mass spectrometry (ToF–SIMS, ION-ToF) was conducted on the same electrodes used for XPS analysis after formation with 100-100-1 μm^3^ volume.

### Calculation Details

The Li-ion-binding energy was calculated using ORCA 4.2.1, employing geometry optimization with the B3LYP functional and the triple-zeta valence polarization basis set. The cation-binding energy was determined using the following equation:

$${\text{Cation-binding energy}} = E_{{{\text{Li}} - {\text{ion}}}} + E_{{{\text{anion}}}} - E_{{{\text{SEI}}}}$$

### Differential Electrochemical Mass Spectrometer Analysis

Gas analysis was conducted using an *in situ* differential electrochemical mass spectrometer (HPR-40 DEMS, Hiden Analytical). Gas components were analyzed during the initial de-sodiation of the O3-type positive electrode half-cell between the open-circuit voltage of 4.0 V (vs. Na/Na^+^) at a rate of 0.1 *C*. The gas evolution of the cells was measured at 10-min intervals.

### Transmission Electron Microscopy Analysis

HR-TEM analysis of the cycled O3-type positive electrode was performed with JEM-ARM200F (JEOL, Japan) after FIB-based sample preparation. The EELS map was obtained from the reconstruction of EELS spectra from the surface of the cycled O3-type positive electrode.

### Electrochemical Quartz Crystal Microbalance Test

The sealed well-type electrochemical quartz crystal microbalance (EQCM) cell with the Na/Pt-sputtered resonator (QA-A9M, SEIKO EG&G) was applied to compare the mass change of the electrode with background and LiPF_6_-added electrolytes. A scan rate for linear sweep voltammetry was 1.0 mV s^−1^ at the voltage range of the open-circuit voltage (OCV) to 0.7 V (vs. Na/Na^+^), and the electrode mass was calculated based on the Sauerbrey equation with measured resonance frequency change.

## Results and Discussion

### Electrolyte Characterization and Fundamental Understanding of Li Salt Effect

**Fig. 1:**
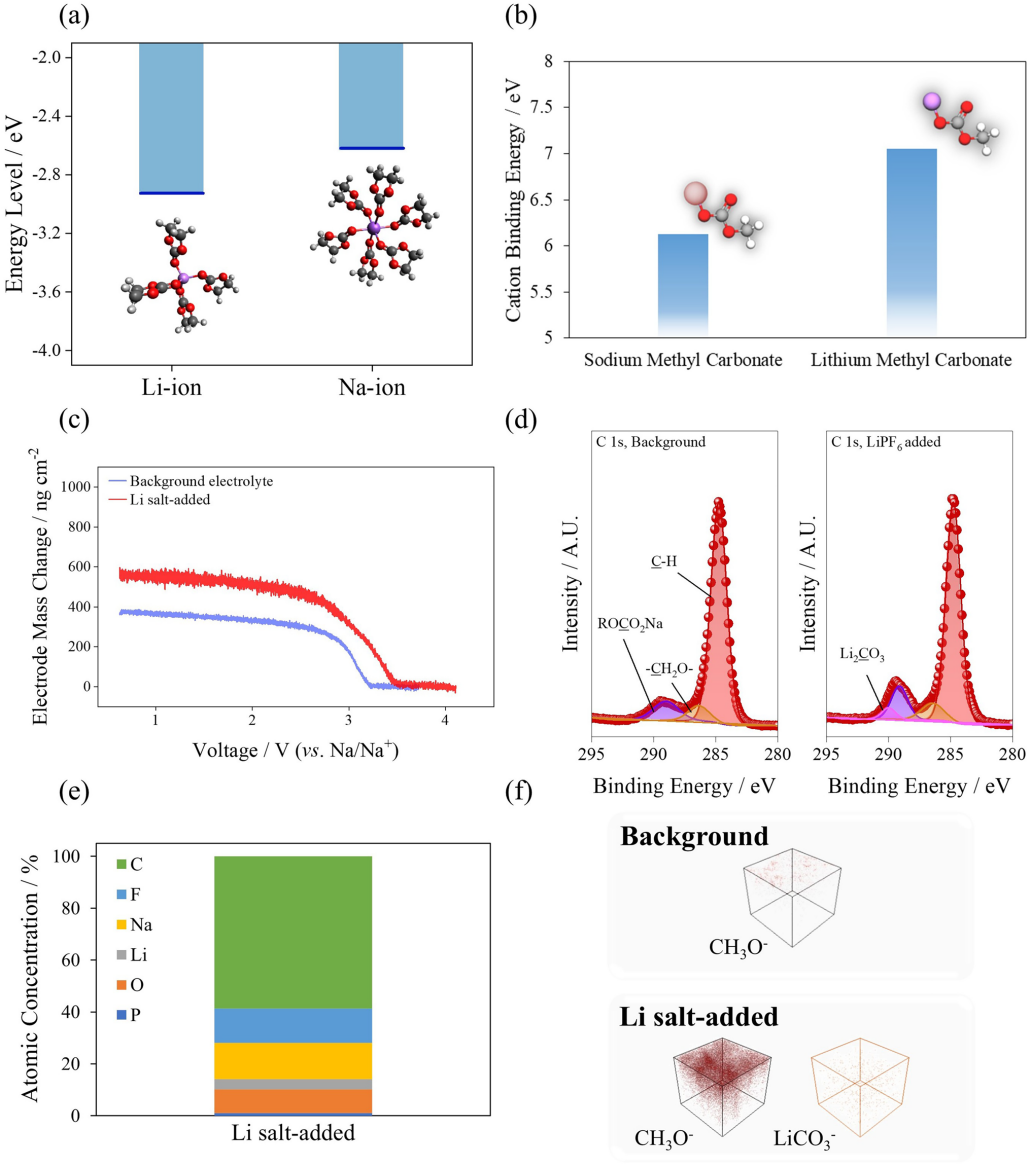
**a** Lowest unoccupied molecular orbital energy levels of Li- and Na-solvated clusters, **b** cation-binding energy of the typical solid electrolyte interphase components in sodium and Li-ion batteries, *ex-situ* **c** voltage vs. electrode mass change profile obtained from the EQCM measurement, **d** C 1*s* X-ray photoelectron spectra, **e** atomic concentration and **f** three-dimensionally reconstructed time-of-flight secondary ion mass spectroscopy signal from background and the added Li salt electrolytes, respectively

Figure 1 shows the effect of incorporating Li salt (in the SIB electrolyte) on the interphase formation on the negative electrode surface. Figure 1a depicts the lowest unoccupied molecular orbital (LUMO) energy levels of the Li- and Na-ion-bound ethylene carbonate (EC) clusters because LUMO levels constitute the indicator for reducibility of electrolytes. It is worthwhile to note that the introduction of Li salt in the electrolyte forms Li–EC solvation cluster, which is based on the four EC-bound Li ion from Raman spectra (Fig. S1). The LUMO energy level of the Na–EC cluster was lower than that of the Li–EC cluster, and improved SEI formation kinetics were expected following the addition of a Li salt. The linear sweep voltammetry (LSV) test was performed to compare the reductive decomposition onset of background and LiPF_6_-added electrolytes (Fig. S2). The voltammogram shows the onset voltage for cathodic decomposition of electrolyte is increased with LiPF_6_ application; hence, the shift of LUMO energy level by incorporation of LiPF_6_ salt was cross-examined by voltammetry test [41]. The developed current density from the Li salt incorporation is increased than that of background electrolyte, which is only composed with Na ions. This implies that the SEI formation kinetics is improved with Li salt introduction. Furthermore, the cation-binding energies of typical Na- and Li-ion-bound SEI components were compared (Fig. 1b). The cation-binding energy increased with Li-ion inclusion in SEI, implying that the chemical stability of the SEI component in the electrolyte improved with Li-SEI formation because low-cation-binding energy considerably increased the solubility of the SEI component [30, 42, 43]. The incorporation of an additional Li salt in the electrolyte led to the marginal decrement of ionic conductivity of the electrolyte (Fig. S3). The electrochemical quartz crystal microbalance (EQCM) evaluation of background and additive-added electrolytes, respectively, was performed to evaluate mass increment from facilitated SEI film deposition during the initial negative voltage exposure (Fig. 1c). The voltage versus electrode mass change profile evidently shows that the increase in the electrode mass is occurred significantly at higher voltage of electrode with Li salt-added electrolyte than that of background electrolyte. Note that the applied electrode was platinum-coated resonator, and thus, the tendency in electrode mass change can be compared. The spectroscopic characterization of SEI formed on hard carbon electrode with background and Li salt-added electrolytes was performed. The C 1*s* XPS spectra showed the formation of lithium carbonate species [30, 42, 44, 45] with increased SEI concentration on the hard carbon at the instant of Li salt addition, and the atomic concentration of the Li ion in the SEI was 3.9% (Fig. 1d, e). Hence, the addition of Li salt facilitates the formation of SEI on hard carbon electrodes, ensuring a decrease in the cathodic side reaction on hard carbon electrodes. The three-dimensional time-of-flight secondary ion mass spectroscopy (3D ToF–SIMS) maps (Fig. 1f) show an increased SEI deposition following the addition of Li salt and the lithium carbonate formation in the inner layer of the SEI. As the LUMO of Li–EC was lower than that of Na–EC, the preferential reduction of Li–EC was predictable from thermodynamic assessments. The initial reduction in the Li–EC cluster formed the inner Li_2_CO_3_ layer on hard carbon and was subsequently observed in the 3D ToF–SIMS map.

**Fig. 2:**
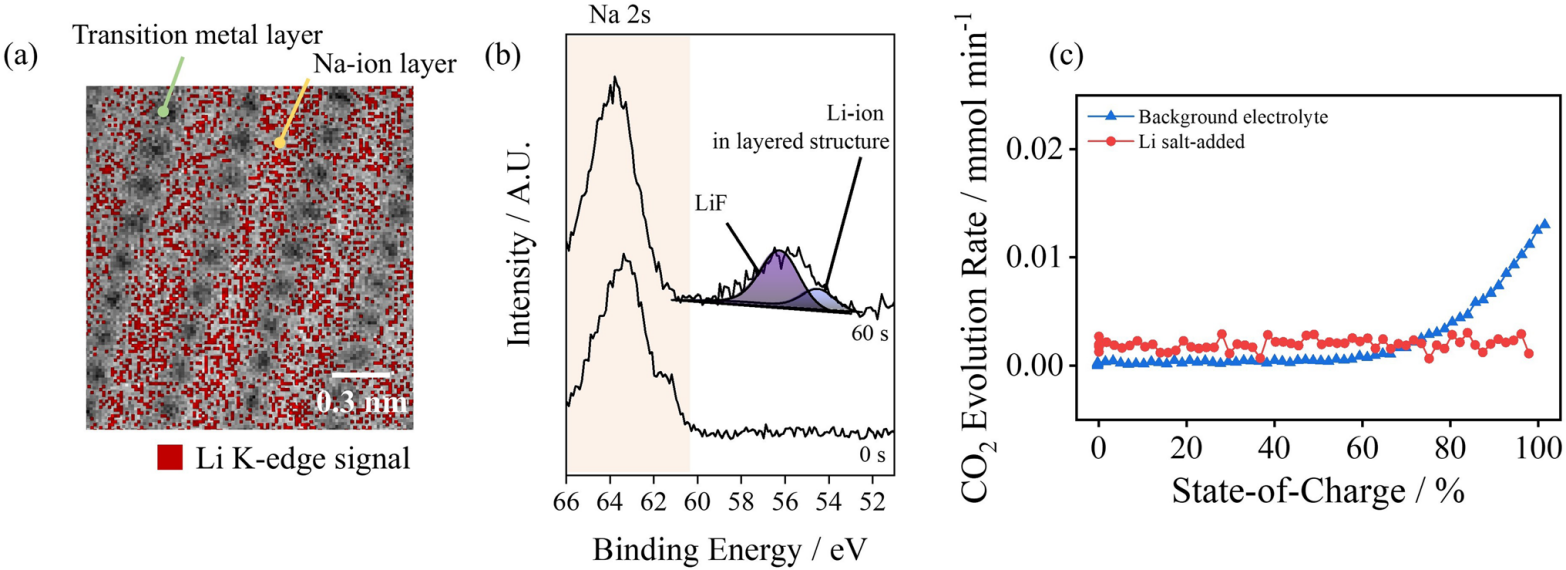
**a** Li K-edge electron energy loss spectroscopy signal map obtained from the focused ion beam-processed high-resolution transmission electron microscopy analysis after formation, **b** *postmortem* Na 2*s* and Li 1*s* X-ray photoelectron spectra obtained from the addition of the Li salt electrolytes, cycled O3-positive electrode, and **c** gas evolution profile recorded using the differential electrochemical mass spectrometry analysis

The surface characteristics and subsequent electrochemical performances of the O3-positive electrode are depicted in Fig. 2. Figure 2a shows the Li K-edge signal mapping results obtained from the focused ion beam (FIB)-processed, high-resolution transmission electron microscopy (HR-TEM) analysis. The obtained Li K-edge electron energy loss spectroscopy (EELS) peaks were processed to demonstrate the Li occupation site at the O3-type, positive electrode structure. The Li K-edge mapping result shows that Li ions occupied the Na-ion layer at the surface of the O3-positive electrode, implying that the incorporation of Li salt in the electrolyte led to the lithiation of the O3-positive electrode surface. Studies have shown that the doping of the Li ion in Na-intercalated layer at the O3-type positive electrode improves the structural stability from the pillar formation [16, 18]; thus, surface stabilization is expected from this surface intercalation. The intercalated Li ion in the Na-ion layer serves as pillar for the transition metal layer during cycling, which suppresses the oxygen release and the lattice collapse from the structural instability. The ToF–SIMS profile of the O3-positive electrode after formation (Fig. S4) showed the LiO^−^ signal after etching of surface film, indicating the marginal lithiation of the positive electrode following the incorporation of LiPF_6_, as evidenced by the TEM analysis. *post-mortem* XPS analysis revealed the formation of LiF and the intercalated Li in the layered structure at the surface of O3-type positive electrode [46–49]; thus, a structural reinforcement from the *in situ* surface doping was expected (Fig. 2b). The gas evolution quantified using differential electrochemical mass spectrometry (DEMS) indicated that the CO_2_ gas evolution from the anodic side reaction at the surface of the positive O3 electrode was effectively suppressed with the incorporation of the Li salt additive (Fig. 2c). Note that the state-of-charge 0 (SOC 0) and SOC 100 denote the open-circuit voltage and 4.0 V (vs. Na/Na^+^), respectively (Fig. S5). The gas evolution rate was highly mitigated following the incorporation of the additive; this effect is attributed to the surface stabilization by the Li pillar at the surface and the highly passivating LiF formation. The half-cell cycle performance of the Na metal/O3-type positive electrode led to a considerable improvement in capacity retention, i.e., 46.5% at the 100th cycle, following the addition of LiPF_6_, indicating that the reinforcement of the O3-type electrode surface is sufficiently achieved by introducing the additive (Fig. S6).

The hard carbon/sodium half-cell also demonstrates the stable cycle performance with improved Coulombic efficiency with LiPF_6_ introduction with 0.01–1.5 V (vs. Na/Na^+^) voltage range (Fig. S7). Meanwhile, the cycleability evaluation at half-cell configuration for hard carbon electrode is not ideal because the growth of resistance from the sodium metal significantly affects the cycle performance because the majority of capacity from hard carbon electrode is delivered from low-voltage plateau region, which is highly affected by sodium metal resistance.

**Fig. 3:**
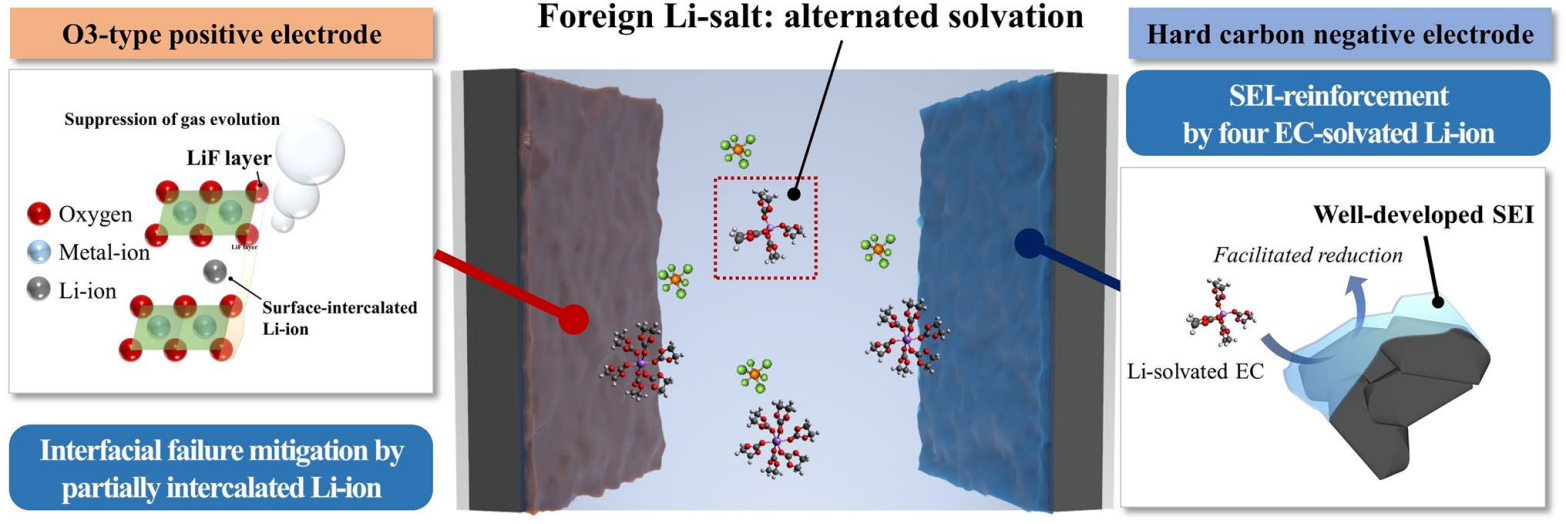
Scheme showing the working mechanism of LiPF_6_ in sodium-ion batteries with a hard carbon O3 configuration

The effects of LiPF_6_ on hard carbon O3 electrode-based SIBs are illustrated in Fig. 3. At the surface of the negative electrode, the incorporation of LiPF_6_ altered the microsolvation structure. The Li ion preferred solvation with four EC molecules that enable facile reduction compared with the background electrolyte with only sodium salts. From the negatively shifted LUMO level of the solvated Li ion, the passivation ability of the SEI was highly reinforced by the preferred reduction of the Li-solvated cluster. Furthermore, the Li_2_CO_3_ formation in the inner SEI layer suppressed film dissolution, which is regarded as a typical failure for the SIBs because the cation-binding energy increases with the Li-ion-based SEI component compared with that of Na-ion-based SEI. On the contrary, marginal surface doping and LiF formation on the O3-positive electrode surface were achieved following the introduction of the Li salt. As the Li ion serves as a pillar ion for the O3-positive electrode, the release of oxygen from the surface was highly mitigated. The CO_2_ gas evolution at high-voltage exposure decreased significantly with electrolyte formulation, which is evidenced by DEMS analysis. Consequently, the deterioration of the positive electrode was highly suppressed with the surface fortification, which enabled a major increment in the capacity retention of the O3-positive electrode-based half-cells.

### Electrochemical and Spectroscopic Characterization of Li Salt-Added Electrolyte in Pouch Cells

**Fig. 4:**
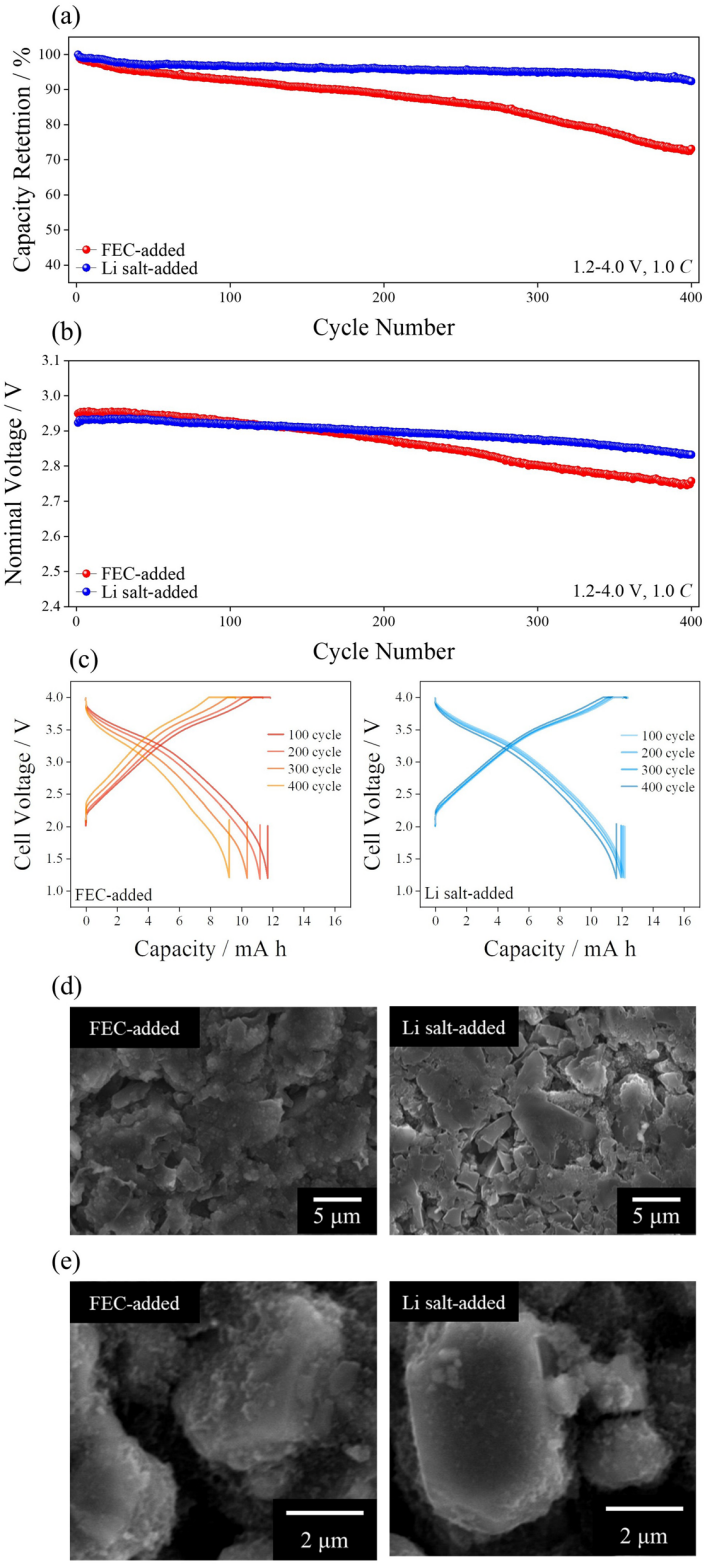
**a** Capacity retention, **b** nominal working voltage, and **c** cycle number-dependent voltage profile of hard carbon/O3-type positive electrode-based pouch cell. *ex-situ*, top-view scanning electron microscopy images of **d** hard carbon electrodes and **e** O3-type positive electrodes after 400 cycles

LiPF_6_ additive was incorporated in the pouch cell composed of the hard carbon/O3-type positive electrode and evaluated (Fig. 4a). The addition of the LiPF_6_ salt in the electrolyte improved capacity retention considerably compared with that of the typical FEC-added electrolyte. While the FEC-added pouch cell exhibited a capacity retention of 73.1% at the 400th cycle, the addition of LiPF_6_ salt in the SIB electrolyte demonstrated a capacity retention of 92.7% at the same cycle number. This achieved capacity retention is highly surpasses previously reported results, which is typically about 80% (Table S1). The working nominal voltage change during the cycle indicates that the development of polarization from side reactions occurred in the case of the FEC-added cell (Fig. 4b). The working voltage with the LiPF_6_-added electrolyte was highly preserved in different cycles, implying that the energy delivered from the addition of the LiPF_6_ pouch cell was well retained after the 400th cycle. Figure 4b demonstrates the cycle number-dependent voltage profiles obtained from hard carbon/O3-type positive electrode pouch cells. While polarization increased during repeated cycling in the FEC-added cell case, the LiPF_6_-added cell showed a stable voltage profile even after the 400th cycle. The improved Coulombic efficiency was observed at pouch cell with LiPF_6_-added electrolyte than that of FEC-added electrolyte-injected cell (Fig. S8).

Figure 4c, d shows the SEM images from the cycled hard carbon electrodes and O3-type positive electrodes that were dismantled from the cycled pouch cells. The hard carbon electrode cycled with the FEC-added electrolyte generated thick deposits on the surface from additional electrolyte decomposition during the various cycles. By contrast, the introduction of the LiPF_6_ salt inhibited further deposition of SEI on the as-formed SEI because a less soluble and stable SEI was deposited after the formation process from the readily reducible, Li-solvated EC cluster. The C 1*s* XPS spectra also implied that the additional SEI deposition was suppressed following the addition of LiPF_6_ after cycling (Fig. S9). Energy-dispersive spectroscopic analysis at the corresponding SEM image revealed an increased concentration of oxygen species at the hard carbon electrode cycled with FEC-added electrolyte, while the cycled hard carbon with LiPF_6_-added electrolyte demonstrated a restrained increment in the oxygen species, which could be attributed to the reduction in carbonate electrolytes (Fig. S10). The additional deposition of the surface film was hindered following the addition of Li salts; hence, the SEM image of the cycled O3-type positive electrode exhibits a rather clean surface in the case of the Li salt-added electrolyte compared with the FEC-added electrolyte case (Fig. 4d). Thus, the oxidative decomposition of electrolytes on the surface of the positive electrode was well suppressed following the addition of LiPF_6_. While LiPF_6_ reinforced the interface and subsequently improved cycleability, the addition of LiODFB in the electrolyte was not beneficial because a thick SEI was deposited on the surface of the hard carbon electrode following the vigorous decomposition of LiODFB (Fig. S11). The initial polarization of LiODFB-added electrolyte was higher than that of LiPF_6_-added electrolyte owing to the excessive SEI formation from LiODFB (Fig. S12).

**Fig. 5:**
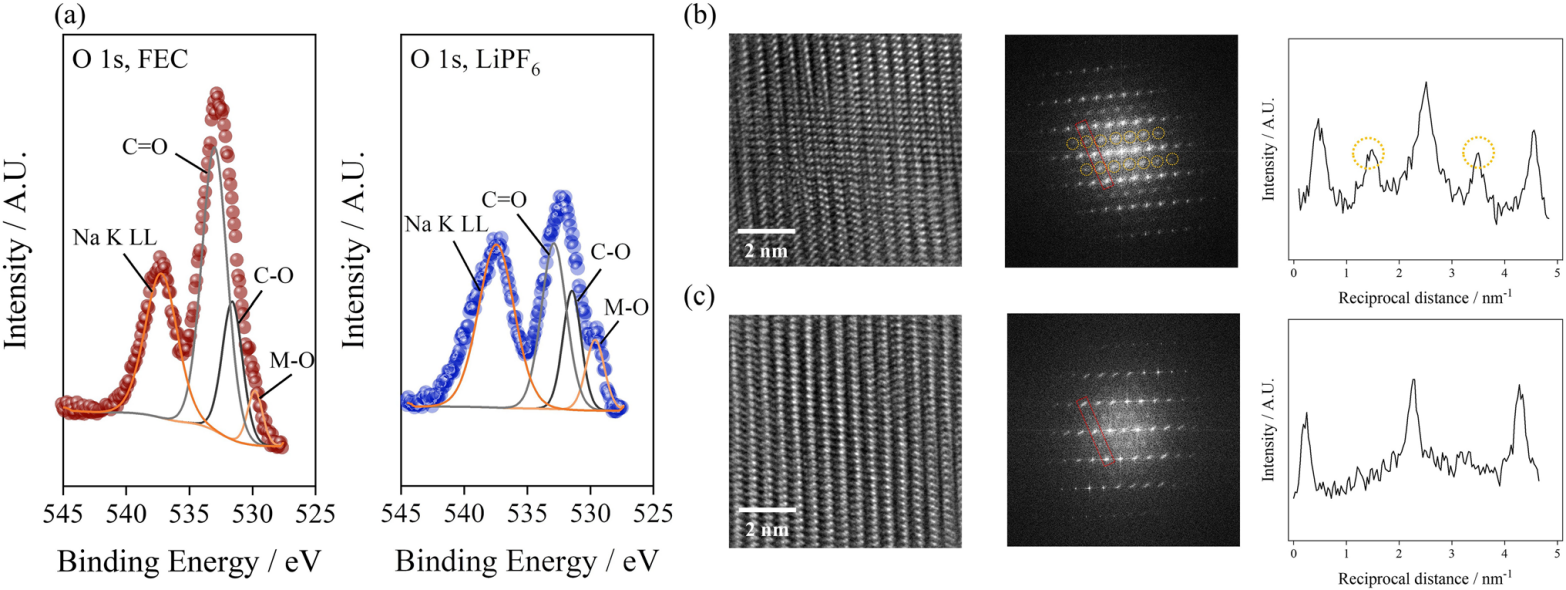
**a** Topmost O 1*s* X-ray photoelectron spectra obtained from cycled O3-type positive electrodes, HR-TEM image and corresponding of fast Fourier transformed image and intensity profile of red dotted line at zone axis of [1 0 0] from 400th cycled O3-type positive electrode with **b** FEC-added and **c** Li salt-added electrolytes, respectively

Figure 5 demonstrates the surface chemical structural failure analysis after 400th cycles of the positive electrode, using XPS and high-resolution transmission electron microscopy (HR-TEM). Figure 5a demonstrates O 1*s* narrow scanned spectra recorded from the 400th cycled O3-type positive electrodes. The O3-type positive electrode cycled with FEC additive illustrates the thick deposition of electrolyte decomposed products (C=O, C–O) and the subsequent surface shrouding of the positive electrode by surface film, as evidenced by the weak lattice oxygen signal. By contrast, the O 1*s* XPS spectra with LiPF_6_-added electrolyte indicate the suppressed deposition of the surface film, which can be deduced from the evident lattice oxygen signal. The deterioration of the surface of the O3-type positive electrode occurs with the formation of spinel and rock salt phases at the surface from collapse of layered structure due to the migration of transition metal ion toward Na-ion layer [15, 17]. Hence, *ex situ* HR-TEM and subsequent fast Fourier transformed (FFT) image analysis were performed on the O3-type positive electrode after 400th cycle. Note that the HR-TEM analysis was conducted at the surface of positive electrodes, implied that the comparative analysis of interface failure can be conducted with HR-TEM analysis. With FEC-added electrolyte, the degradation of surface is depicted, which is evidenced by spinel phase formation at the surface. In contrast, the layered structure is well preserved with Li salt addition from the mitigated interfacial failure of O3-type positive electrode. As can be observed from intensity profiles of FFT patterns at Fig. 5b, c, additional peaks are observed, which are formed by spinel-like phase due to cation disordering from surface degradation. The reciprocal distance profiles at red square shows the additional peak at cycled positive electrode with FEC-added electrolyte, while the LiPF_6_-added electrolyte demonstrates no signal from the spinel phase formation. Since surface-intercalated Li ions effectively stabilize the surface of the O3-positive electrodes, the formation of spinel species due to surface structural degradation is significantly suppressed, which implies that interfacial degradation is substantially suppressed by surface-intercalated Li ions.

By comparing the cycle performance with postmortem analysis with background electrolyte with LiPF_6_-added electrolyte, the significant enhancement of cycleability was demonstrate by LiPF_6_ addition (Fig. S13a). While the spinel phase formation (Fig. S13b) and thick SEI film deposition (Fig. S13c) at positive and negative electrode was demonstrated from background electrolyte, respectively, the Li salt incorporation highly improves the interfacial stability from reinforcement of electrolyte–electrode interfaces. The FFT image from the cycled O3-type positive electrode with background electrolyte shows the dot from the spinel phase formation, which is from the interfacial failure during cycle. Further the deposition of thick SEI was observed with background electrolyte than that of LiPF_6_-added electrolyte due to the vulnerable SEI deposition on hard carbon surface from background electrolyte. Further, the rate capability evaluation with background and Li salt-added electrolytes shows no discharge capacity fading from marginal ionic conductivity decrease by Li salt incorporation because the demonstrated ionic conductivity value was high (Fig. S13d).

## Conclusions

The incorporation of the LiPF_6_ salt in the SIB electrolyte reinforced considerably both the interfaces of the positive and negative electrodes owing to the respective robust SEI and pillar formation at the surfaces of the hard carbon and O3-type positive electrodes. The formation of highly passivating SEI on the hard carbon electrode suppressed additional Na-ion and electron leakages owing to additional electrolyte decomposition, which improved cycle performances. The introduction of Li ions in the electrolyte changed the solvation cluster. The four EC molecules were bound by the Li ions; hence, the Li-solvated cluster can be readily reduced as compared with the Na-solvated cluster case, thus allowing the formation of SEI. As the Li-ion SEI film was less soluble in carbonate electrolytes owing to the smaller cation size than compared with the size of the Na ion, the dissolution of the SEI was less predictable from the Li-contented SEI. Hence, the passivation ability of SEI improved following the addition of the LiPF_6_ salt. By contrast, the marginal surface intercalation of the Li ion at the surface of the O3-positive electrode greatly reduced the oxygen-release-based, induced-gas evolution. Moreover, the formation of the LiF layer on the O3 surface reduces further electrolyte decomposition. A simultaneously reinforced interface improves capacity retention significantly compared to that of the typically applied additive FEC. The achieved capacity retention at the monolayered pouch cell of the hard carbon/O3-type positive electrode SIB configuration following LiPF_6_ salt addition was 92.7% after 400 cycles.

## Supplementary Information

Below is the link to the electronic supplementary material.

Supplementary file1 (DOCX 2190 KB)
