# Supplementary material for: Transformative Effect of Li Salt for Proactively Mitigating Interfacial Side Reactions in Sodium-Ion Batteries
Source: Nanomicro Lett. 2025 Apr 21;17:226. doi: 10.1007/s40820-025-01742-z (PMC12011690; doi:10.1007/s40820-025-01742-z)
Supplement: Supplementary file 1 — Supplementary file1 (DOCX 2190 KB) [file 40820_2025_1742_MOESM1_ESM.docx]

Supporting Information for

**Transformative Effect of Li Salt for Proactively Mitigating Interfacial Side Reactions in Sodium-Ion Batteries**

Jooeun Byun ^1,#^, Joon Ha Chang ^2,#^, Chihyun Hwang ^1,#^, Chae Rim Lee ^1^, Miseung Kim ^1^, Jun Ho Song ^1^, Boseong Heo ^3^, Sunghun Choi ^3^, Jong Hyeok Han ^4^, Hee-Jae Jeon ^5^, Beom Tak Na ^6^, Youngjin Kim ^3,^*, Ji-Sang Yu ^1,^*, and Hyun-seung Kim ^1,^*

^1^ Advanced Batteries Research Center, Korea Electronics Technology Institute, 25, Saenari-ro, Seongnam 13509, Republic of Korea

^2^ Analysis and Assessment Research Group, Research Institute of Industrial Science and Technology, Cheongam-ro 67, Pohang 37673, Republic of Korea

^3^  Department of Battery Convergence Engineering, Kangwon National University1, Kangwon-daehakro, Chuncheon-si, Kangwon 24341, Republic of Korea

^4^ Department of Mechanical and Biomedical Engineering, Kangwon National University 1, Kangwon-daehakro, Chuncheon-si, Kangwon 24341, Republic of Korea

^5^ Department of Smart Health Science and Technology, Kangwon National University 1, Kangwon-daehakro, Chuncheon-si, Kangwon 24341, Republic of Korea

^6^ Research Institute of Intelligent Manufacturing & Materials Technology, Korea Institute of Industrial Technology, Gaetbeol-ro 156, Incheon, Republic of Korea

^#^Jooeun Byun, Joon Ha Chang, and Chihyun Hwang contributed equally to this work.

*Corresponding authors. E-mail: [ykim@kangwon.ac.kr](mailto:ykim@kangwon.ac.kr) (Youngjin Kim); [jisang@keti.re.kr](mailto:jisang@keti.re.kr) (Ji-Sang Yu); [hskim0113@keti.re.kr](mailto:hskim0113@keti.re.kr) (Hyun-seung Kim)

**Supplementary Figures and Table**





**Fig. S1** Raman spectra obtained from background and LiPF_6_-added electrolytes, respectively



**Fig. S2** Linear sweep voltammogram obtained from Cu/Na cell with background and LiPF_6_-added electrolytes





Fig. S3 Ionic conductivities of background and LiPF_6_-added electrolytes


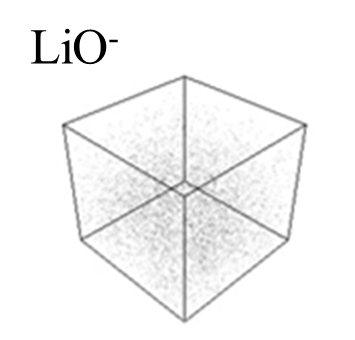


Fig. S4. Three-dimensionally reconstructed time-of-flight secondary ion-mass spectroscopy (ToF-SIMS) map obtained from the O3-type positive electrode cycled with the LiPF_6_-added electrolyte





Fig. S5 First cycle voltage profile of the O3-type positive electrode in a sodium half-cell


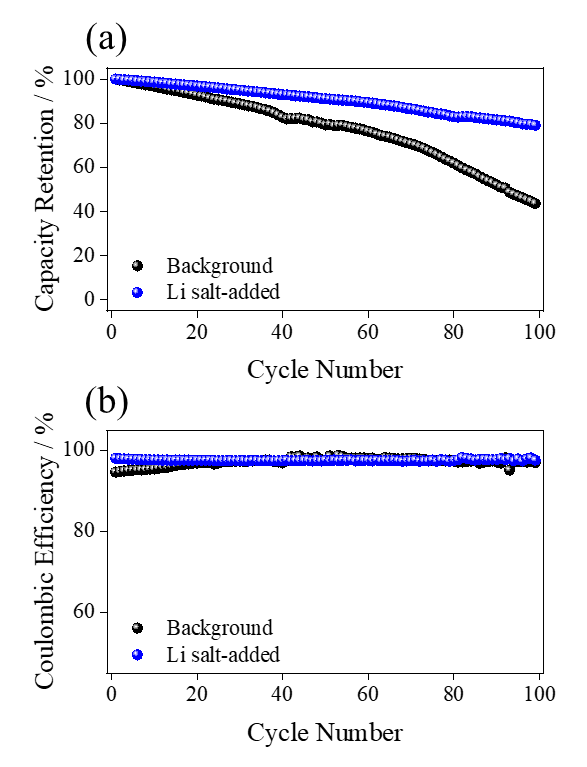


Fig. S6 (a) Cycleability and (b) Coulombic efficiency obtained from the O3 electrode-based half-cells with background and Li salt-added electrolytes


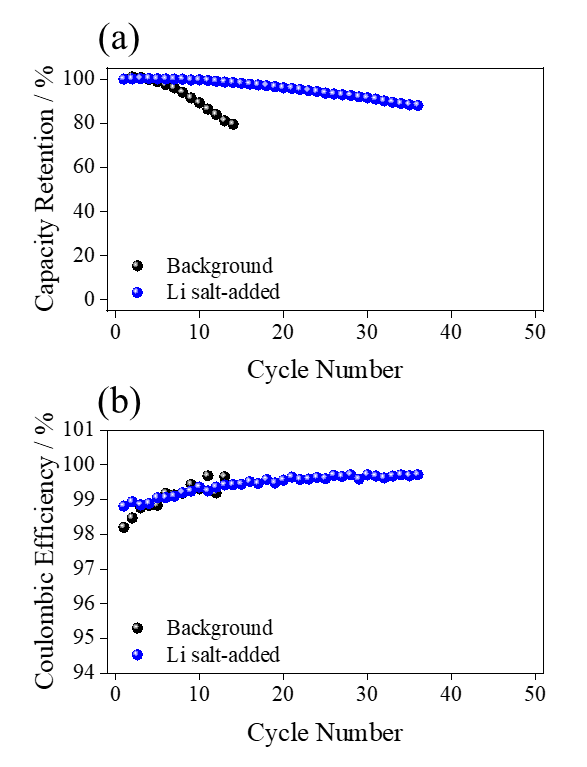


Fig. S7 (a) Cycleability and (b) Coulombic efficiency obtained from the hard carbon electrode-based half-cells with background and Li salt-added electrolytes





Fig. S8 Coulombic efficiency obtained from the hard carbon/O3-type electrode based pouch-cells with FEC-added and Li salt-added electrolytes, respectively


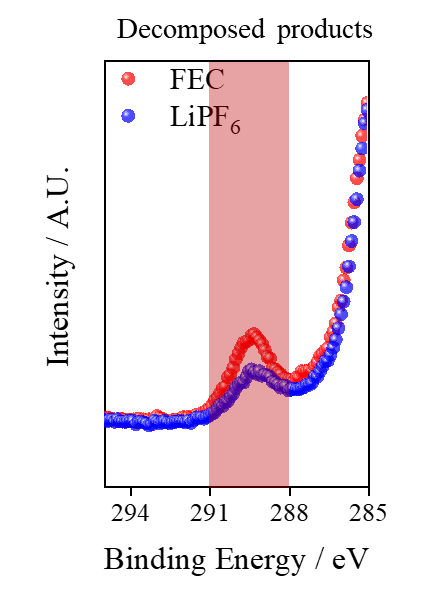


**Fig. S9** C 1s X-ray photoelectron spectra obtained from the hard-carbon electrode after 400 cycles with the addition of the FEC and LiPF_6_ electrolytes


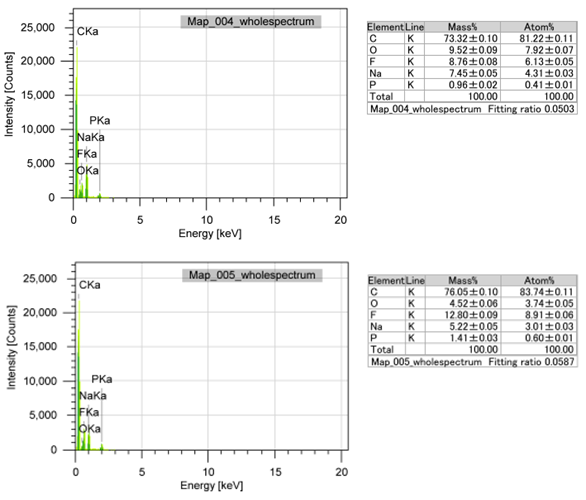


**Fig. S10** Energy-dispersive spectroscopic results recorded from the cycled hard-carbon electrodes following the addition of the FEC and LiPF_6_ electrolytes


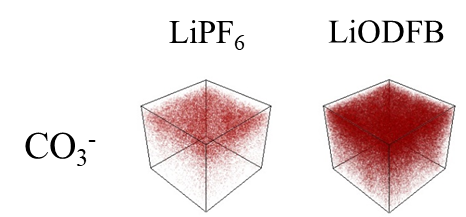


**Fig. S11** Three-dimensionally reconstructed ToF-SIMS map of the hard-carbon electrode after formation following the addition of the LiPF_6_ and lithium difluoro(oxalato)borate (LiODFB) electrolytes





**Fig. S12** Initial voltage profiles after the formation at pouch cells with the LiPF_6_ and LiODFB electrolytes


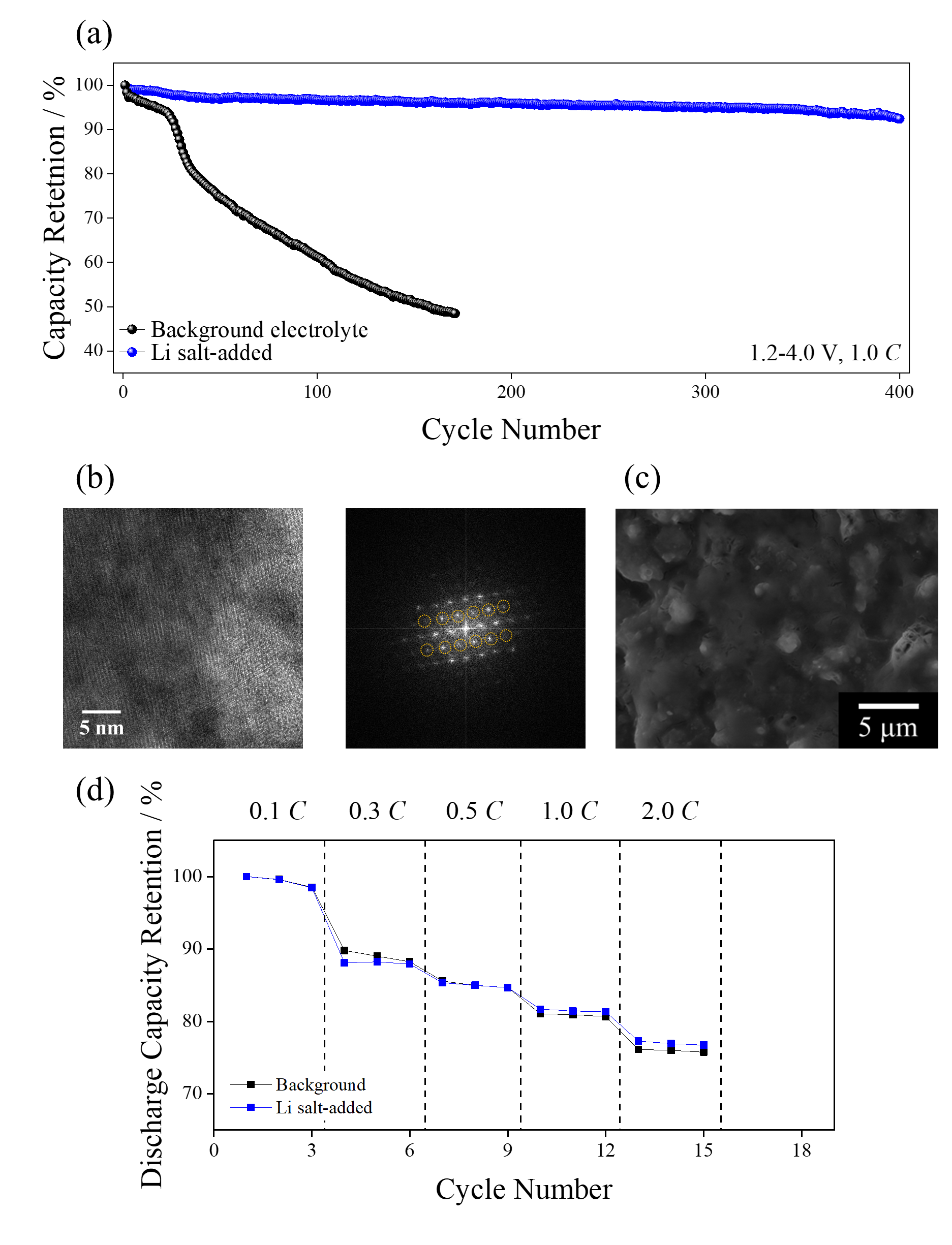


**Fig. S13** (**a**) cycle performance obtained from background, FEC-added, and Li salt-added electrolytes, respectively, (**b**) HR-TEM and corresponding FFT images of cycled positive electrode, (**c**) SEM image of cycled negative electrode, (**d**) rate capability obtained from pouch cells with background and Li salt-added electrolytes

**Table S1** The acheived capacity retention from recently reported literature and this work with hard carbon/O3 electrodes configuration

|  | **Voltage Range  / V** | **Cycle Number** | **Retention / %** | **Mass loading  / mg cm^-2^** | ***C*-rate / *C*** | **References** |
| --- | --- | --- | --- | --- | --- | --- |
| 1 | 1.5-4.0 | 170 | 96 | 1.6 | 0.3 | Chemical Engineering Journal,  2024, 491, 151949 |
| 2 | 2.0-4.0 | 500 | 81 | 2.0 | 0.2 | Small, 2024, 2407425 |
| 3 | 1.5-4.0 | 16 | 80 | 4.3 | 0.2 | Electrochimica Acta, 2018, 281, 370 |
| 4 | 1.9-3.9 | 200 | 85 | 1.0 | 1.0 | Energy Storage Materials,  2025, 74, 103894 |
| 5 | 1.5-4.2 | 100 | 68 | N.A. | 1.0 | Journal of Alloys and Compounds, 2023, 968, 171808 |
| 6 | 1.0-4.0 | 200 | 77 | 1.0 | 1.0 | Journal of The Electrochemical Society, 2023, 170, 070518 |
| **7** | **1.2-4.0** | **400** | **92.7** | **15.0** | **1.0** | **This Work** |
